# Applied One Health: Nigeria National Veterinary Research Institute COVID-19 pandemic response

**DOI:** 10.4102/ojvr.v91i2.2156

**Published:** 2024-09-04

**Authors:** Clement A. Meseko, Ismaila Shittu, Olayinka O. Asala, Adeyinka J. Adedeji, Tinuke A. Laleye, Ebere R. Agusi, Dorcas A. Gado, Kayode A. Olawuyi, Nicodemus Mkpuma, Chinyere Chinonyerem, Bitrus Inuwa, Nneka Chima, Ruth Akintola, Patrick Nyango, Hellen Luka, Judith Bakam, Rebecca Atai, Dennis Kabantiyok, Mark Samson, ThankGod Daniel, Joshua Oyetunde, Olajide A. Owolodun, David D. Lazarus, Emmanuel T. Obishakin, Pam D. Luka, Benshak J. Audu, Sunday Makama, Hussaini G. Ularamu, Yiltawe S. Wungak, James S. Ahmed, Reuben A. Ocholi, Maryam Muhammad

**Affiliations:** 1 Department of Infectious and Transboundary Animal Diseases (ITADs), National Veterinary Research Institute, Vom Plateau State, Nigeria; 2 Department of Quality Assurance, National Veterinary Research Institute, Vom Plateau State, Nigeria; 3 Department of Vaccine Production, National Veterinary Research Institute, Vom Plateau State, Nigeria; 4 Department of Bacteriology, Parasitology and Virology, National Veterinary Research Institute, Vom Plateau State, Nigeria; 5 Department of Biochemistry, Biotech and Drug Development, National Veterinary Research Institute, Vom Plateau State, Nigeria; 6 Department of Diagnostic Services, National Veterinary Research Institute, Vom Plateau State, Nigeria

**Keywords:** COVID-19 pandemic, SARS-CoV-2, diagnosis, NVRI, One Health

## Abstract

The COVID-19 pandemic has caused the death of 7.1 million people worldwide as of 7 July 2024. In Nigeria, the first confirmed case was reported on 27 February 2020, subsequently followed by a nationwide spread of SARS-CoV-2 with morbidity and mortality reaching 267 173 and 3155, respectively, as of 7 July 2024. At the beginning of the pandemic, only a few public health laboratories in Nigeria had the capacity for SARS-CoV-2 molecular diagnosis. The National Veterinary Research Institute (NVRI), already experienced in influenza diagnosis, responded to the public health challenge for the diagnosis of COVID-19 samples from humans. The feat was possible through the collective utilisation of NVRI human and material resources, including biosafety facilities, equipment, reagents and consumables donated by international partners and collaborators. Within 6 months of the reported COVID-19 outbreak in Nigeria, over 33 000 samples were processed in NVRI facilities covering five states. Thereafter, many field and laboratory projects were jointly implemented between NVRI and collaborating sectors including the Nigerian Centre for Disease Control (NCDC) and the National Institute for Medical Research (NIMR), which brought together professionals in the health, veterinary, education and socio-sciences. In addition, One Health grants were secured to enhance surveillance for coronavirus and other zoonoses and build capacity in genomics. Bio-surveillance for coronaviruses and other emerging zoonotic pathogens at the human–animal interface was activated and continued with sample collection and analysis in the laboratory for coronaviruses, Lassa fever virus and Mpox. One Health approach has shown that inter-sectoral and multinational collaboration for diagnosis, research and development in animals, and the environment to better understand pathogen spillover events at the human–animal interface is an important global health priority and pandemic preparedness.

## Background

The COVID-19 pandemic, caused by severe acute respiratory syndrome coronavirus 2 (SARS-CoV-2), began in Wuhan, China in late 2019 (Phelan, Katz & Gostin 2020). As of 7 July 2024, 776 million people have been infected and 7.1 million deaths recorded worldwide (World Health Organization [WHO] 2024). In Africa, the morbidity and mortality data as of 7 July 2023 were 9.6 million/175 510, while in Nigeria, the number of deaths recorded was 3155 out of 267 173 that were infected as of 15 July 2024 (https://covid19.who.int). Nigeria reported its first confirmed case (imported) on 27 February 2020 followed by a nationwide spread of the SARS-Cov-2 that has been reported in 36 states including the Federal Capital Territory (Abuja) (Agusi et al. 2024).

The initial challenge in the control of COVID-19 was that only a few public health and veterinary laboratories had the capacity and capabilities for SARS-CoV-2 molecular diagnosis. Thus, a One Health approach, as recommended by the WHO and WOAH (formerly OIE), provided evidence of the need for a long-standing and sustainable One Health collaboration, coordination and communication among sectors to control infectious diseases. This approach that takes cognisance of interagency capacity, capability and collaboration, was implemented in Nigeria (https://www.woah.org/en/what-we-offer/emergency-preparedness/covid-19/) during the COVID-19 pandemic management. The National Veterinary Research Institute (NVRI) already with experience in infectious disease control and diagnosis of influenza (Joannis et al. 2008) responded to the public health challenge and made its BSL-3 and BSL-2 laboratories available for diagnostic services. The BSL-3 at NVRI was built and donated to the Federal Government of Nigeria by the Canadian government before the pandemic in 2016. The facility is the hub of the safe handling of clinical samples collected from humans with suspicion of COVID-19 infection. The feat was also made possible by the collective utilisation of NVRI human and material resources including biosafety equipment, reagents and consumables provided by NVRI management and those subsequently donated by international partners and collaborators including the Africa Union, International Atomic Energy Agency (IAEA), Robert Koch Institute (RKI) and Fredrich-Loeffler-Institut (FLI). Here, we analysed and presented the outcome of multidisciplinary, multisectoral collaboration from the perspective of NVRI in a One Health approach.

## Methods

**FIGURE 1:**
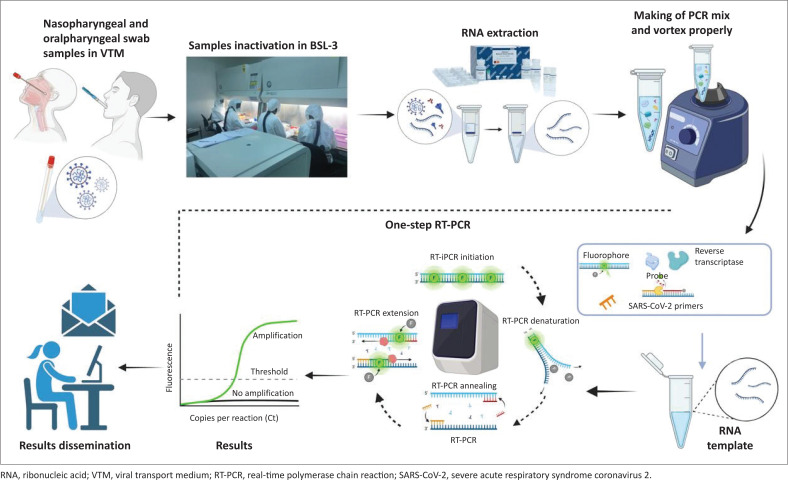
Syndrome coronavirus 2 test algorithm in the BSL-3 and BSL-2 containment facilities at the National Veterinary Research Institute.

**FIGURE 2:**
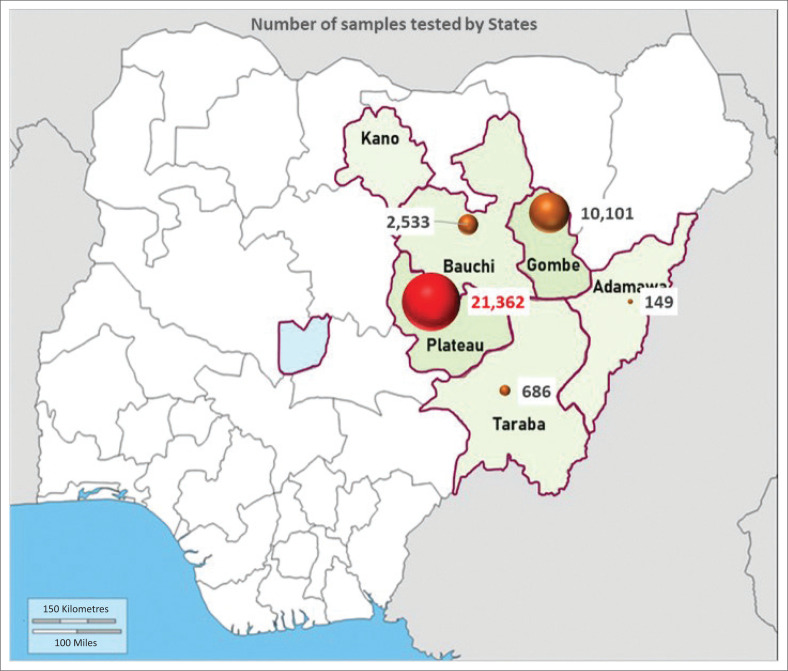
Map of Nigeria showing number of samples tested by state for COVID-19 at the National Veterinary Research Institute, Vom.

The Nigeria Centre for Disease Control and Prevention (NCDC), the national public health agency, on 09 April 2020, requested the NVRI to join the SARS-CoV-2 molecular diagnostic network, which expanded to about 100 laboratories nationwide for human COVID-19 investigation and control. The threat posed by the infectiousness of the hazard made it dangerous even for laboratory personnel to handle the virus unprotected. The National Veterinary Research Institute was able to overcome this challenge through the use of its BSL-2 and BSL-3 high containment facilities. Furthermore, laboratory personnel were trained in infection prevention and control (IPC) as a preamble to handling the dangerous pathogen of the public health scare during COVID-19. As part of providing a secondary layer of protection, powered air purifying respirators (PAPR) were used. The PAPRs were provided by the United States Centre for Disease Control and Prevention (US-CDC) and previously used for Mpox animal reservoir investigation (Meseko et al. 2023). The test algorithm (Figure 1) that was also explained as part of the diagnostic refresher course for BSL-2 and BSL-3 laboratory personnel was segmented to higher and lower biological risk to reduce contamination and control of SARS-CoV-2 and other infections. High-risk activities included receiving clinical specimens and inactivation of the virus in the BSL-3 while the lower biological risk activities involved real-time quantitative polymerase chain reaction (RT-qPCR) and assay of extracted SARS-CoV-2 was carried out at the BSL-2 laboratory. This also guided the approach to testing SARS-CoV-2 in an international, interlaboratory ring trial that confirms the feasibility of an extraction-less ‘direct’ RT-qPCR method for reliable detection of SARS-CoV-2 ribonucleic acid (RNA) in clinical samples in which NVRI collaborated as a member of the network (Mills et al. 2022). Virus inactivation and RNA extraction were performed using many protocols (Table 1) depending on available reagents because of the high turnover of samples from the coverage states (Figure 2). For instance, in using DaAn gene protocols and following manufacturer’s instructions (https://en.daangene.com), lysis buffer containing carrier RNA, for example, for one test = 200 µL of lysis solution was added to 4 mL of carrier RNA, for 20 test = 4 mL of the lysis solution was added to 80 mL of carrier RNA provided. An internal standard solution was briefly used to dissolve carrier RNA (dry powder) and mixed with a lysis solution. Thereafter, 200 µL of the samples were added into the 1.5 mL Eppendorf centrifuge tubes, followed by the addition of 200 µL lysis solutions containing Carrier RNA, fastening down the tube cover and vortex for 15 s then centrifuged at full speed for 10 s and then incubated at room temperature for 1 h. The inactivated samples were then sealed and transferred in the cold chain to the BSL-2 laboratory for nucleic acid extraction. The final elution of purified RNA was performed with 50 µL eluent already preheated at 72 °C. Polymerase chain reaction amplification of SARS-CoV-2 Sarbeco gene according to the manufacturer’s protocols in tubes A and B was performed. Amplification result was interpreted as positive if it was less than 40 copies per reaction (Ct) values and negative when above 40 ct values.

**TABLE 1:** The different assays and protocols used for the detection and amplification of specific syndrome coronavirus 2 genes at the National Veterinary Research Institute, Nigeria during the COVID-19 pandemic.

Assay/protocol	Targeted genes	Ct values (+Ve results)	References
GeneFinder	RdRp, N, and E	≤ 40	Gard et al. (2022)
Sansure	N, ORF 1 a/b	≤ 40	Wang et al. (2020), Lu et al. (2020); Hailemariam et al. (2022)
Beijing Genomic Institute	ORF 1 a/b	≤ 40	Wang et al. (2020), Van Kasteren et al. (2020); Hailemariam et al. (2022)
DaAn Gene	N, ORF1 a/b	≤ 40	Wang et al. (2021); Hailemariam et al. (2022)

Note: Please see the full reference list of the article for more information.

Ct, copies per reaction.

## Results and discussion

**FIGURE 3:**
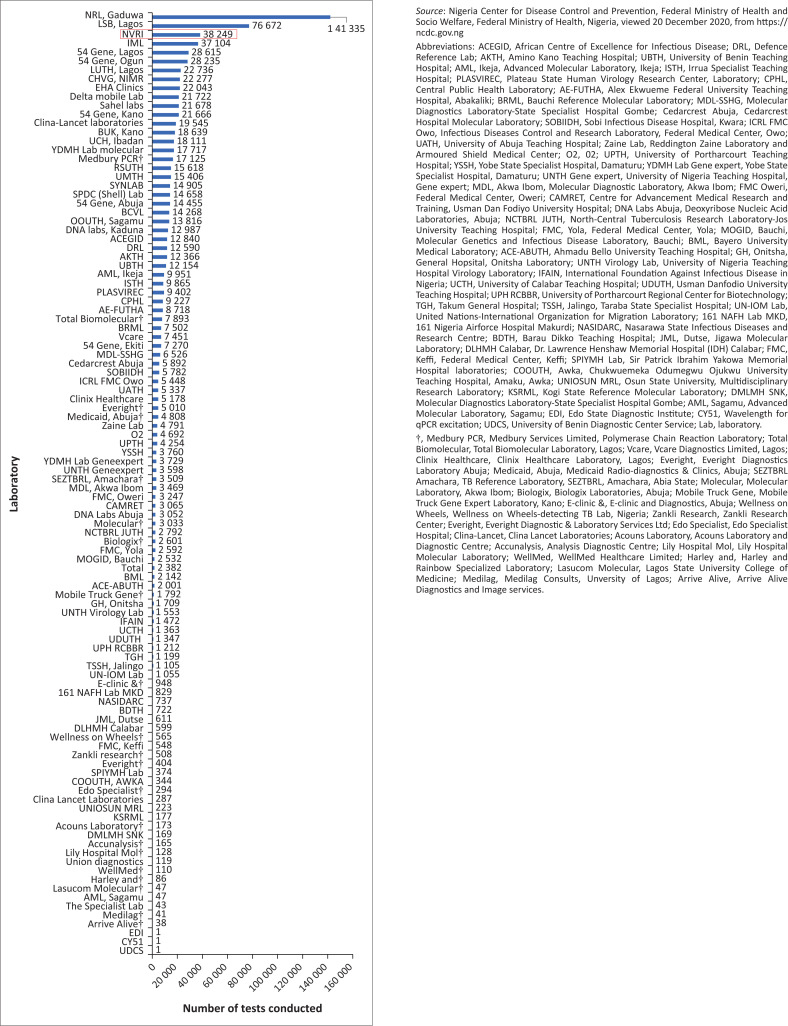
National Veterinary Research Institute (in red) tests of COVID-19 pandemic samples.

Fifty-five thousand human samples were processed by the NVRI between May 2020 and December 2021 out of which 2345 (4.26%) were tested positive by RT-qPCR. In the first 6 months, over 33 000 samples were processed and NVRI performed 10% of the total samples collected ranking 3rd out of 100 laboratories in output by December 2020 (Figure 3). This was possible by the collective utilisation of NVRI human and material resources including a BSL-2 and BSL-3 laboratories. Personal protective equipment, reagents and consumables support came from internal resources at NVRI, and additional support from NCDC, RKI/FLI (Germany), IAEA and AU among others. Many field and laboratory projects were jointly implemented between NVRI and NCDC including knowledge-sharing platforms such as the Nigeria COVID-19 Research Coalition (NCRC) (https://von.gov.ng/tag/the-nigeria-covid-19-research-coalition/). This brought together professionals in the health, veterinary, education and socio-sciences. The Nigeria addressing COVID-19 through One Health (NACOH) research grant was awarded by the Global Health Protection Programme (GHPP) of the German government to build capacity in genomics and achieve the first strands of SARS-CoV-2 sequences at NVRI, Vom (https://ghpp.de/fileadmin/images/ueber-das-ghp/Datasheet_2021/Datasheet_21_CoGLo/NACOH_CGP.pdf).

The National Veterinary Research Institute researchers were actively engaged in incidence control both at the national and state levels and participated in case identification, tracing and sample collection. Collaborations in research are evidenced by a number of publications including the explorative development of antivirals based on Nigerian medicinal plants (Falang et al. 2020; Kabantiyok et al. 2022; Meseko, Shittu & Adedeji 2020).

Enhanced capacity for biosurveillance for coronaviruses and other emerging zoonotic pathogens at the human–animal interface and socio-anthropogenic information were highlighted (Agusi et al. 2024). The NVRI Staff were also actively involved in research communication as evidenced by many other published articles (Agusi et al. 2023, 2024; Forcados et al. 2021; Mills et al. 2022). Nigeria had experienced pandemics before COVID-19 including the Influenza Pandemic of 1918 and Pandemic H1N1 (swine flu) of 2009 (Itodo 2023; Meseko et al. 2019) but none of the previous pandemics had as much impact on the health, economy, livelihood and socio welfare of the people. While pandemics are not predictable, the importance of improving measures for early detection, prevention and mitigation cannot be overemphasised. In Africa generally and Nigeria in particular, the main lessons learnt in the aftermath of the COVID-19 pandemic is the imperative of developing and enhancing in-country capacity for laboratory diagnosis, early detection, reporting and control of the spread of infectious diseases such as SARS-CoV-2. With respect to infectious disease diagnosis, COVID-19 showed similarity and leverage in human and animal health sectors. Mutually beneficial cooperation and collaboration in the sense of the One Health approach and mapping capacity and capability of laboratories in the network can impact disease control. The infectious diseases diagnostic network that was facilitated during the COVID-19 pandemic has the added benefit of improving the diagnostic capacity in Nigeria for many other infections as many molecular diagnostic services are closer to sub-national levels thus improving turn-around time for diagnostic results. Many of these networks of diagnostic laboratories are functional and have expanded beyond clinical diagnosis to include research. Consortium for genomic surveillance, bio-repository and attracting grants for sustainability post-COVID-19 pandemic is a priority within the molecular diagnostic network and is an important contribution to nation-building.

## Conclusion

The importance of One Health collaboration in diagnostics and research in humans and animals in a shared environment cannot be overemphasised. The COVID-19 pandemic provided the opportunity to move from theory to the practice that a long-standing and sustainable One Health collaboration is needed for infectious disease control.
